# Improving Community Health Workers’ Attitudes toward Collaborative Practice in the Care of Older Adults: An In-Service Training Intervention Trial in the Philippines

**DOI:** 10.3390/ijerph18199986

**Published:** 2021-09-23

**Authors:** Kathryn Lizbeth L. Siongco, Keiko Nakamura, Kaoruko Seino, TJ Robinson T. Moncatar, Lourdes Marie S. Tejero, Shelley Ann F. De La Vega, Sheila R. Bonito, Richard Javier, Takako Tsutsui, Yuri Tashiro, Saber Al-Sobaihi, Fely Marilyn E. Lorenzo, Carmelita C. Canila

**Affiliations:** 1 Department of Global Health Entrepreneurship, Tokyo Medical and Dental University, Tokyo 113-8519, Japan; klsiongco@up.edu.ph (K.L.L.S.); seino.ith@tmd.ac.jp (K.S.); tjr.moncatar@gmail.com (T.R.T.M.); ytashi.ith@tmd.ac.jp (Y.T.); saber.tmdu@gmail.com (S.A.-S.); 2 College of Nursing, University of the Philippines Manila, Manila 1000, Philippines; lstejero@up.edu.ph (L.M.S.T.); srbonito@up.edu.ph (S.R.B.); 3 WHO Collaborating Centre for Healthy Cities and Urban Policy Research, Tokyo 113-8519, Japan; 4 College of Public Health, The University of the Philippines Manila, Manila 1000, Philippines; rsjavier@up.edu.ph (R.J.); cccanila@up.edu.ph (C.C.C.); 5 Technology Transfer and Business Development Office, The University of the Philippines Manila, Manila 1000, Philippines; 6 Institute on Aging, National Institute of Health, The University of the Philippines Manila, Manila 1000, Philippines; sfdelavega@up.edu.ph; 7 Human Resource Development Office, The University of the Philippines Manila, Manila 1000, Philippines; 8 Department of Public Health, University of Hyogo, Kobe 651-2197, Japan; chrysanthemum62@yahoo.co.jp; 9 Commission on Higher Education of the Philippines, Diliman, Quezon City 1101, Philippines; marilynlorenzo@gmail.com

**Keywords:** aging society, community health workers, collaborative healthcare, Philippines

## Abstract

The objective of this study was to evaluate the efficacy of an in-service, short-term training program in improving the attitudes toward, and readiness and activities for collaboration among community health workers (CHWs) in a primary care setting in the Philippines. A randomized controlled trial was adopted dividing participants into an intervention (*n* = 42) and a control group (*n* = 39). Attitudes toward, and readiness and activities for collaboration were measured using three standardized scales before and at 6 months after the training. A significant difference (*p* < 0.001) was observed in the Attitudes Toward Health Care Teams Scale (ATHCTS) scores between pre- and post-test in the intervention (6.3 ± 8.3 [Mean ± SD]) and control groups (0.7 ± 8.2). Multivariate linear regression analysis showed an independent positive association between the intervention and greater improvement in the ATHCTS score (Coefficient *β* = 6.17; 95% CI = 0.82, 11.53; *p* = 0.03) at follow-up, after adjustment for age, years in current occupation, and social support role of participants. The results demonstrated the efficacy of the intervention for improving the attitudes of CHWs toward collaborative practice in the care of older adults.

## 1. Introduction

The number of older adults has been rising continuously in the Asia-Pacific region, including the Philippines where senior citizens above 60 years of age accounted for 7.5 percent of the total population in 2015 [1]. The care of older adults is often complex due to longer term illness, multiple comorbidities, and changing living and social circumstances [2]. This complexity of elderly care increases the needs for services from multiple resources and the collaboration among different professions across diverse providers and sectors [3].

The World Health Organization recognizes interprofessional education (IPE) as necessary to achieving a collaborative, practice-ready health workforce composed of health workers who are competent to work in an interprofessional team and are prepared to respond to local and complex health needs [4]. Collaboration in the context of interprofessional teams is widely accepted as being essential to the provision of effective and comprehensive care [5]. The effectiveness of interprofessional learning has been shown to significantly change learners’ reactions, knowledge, skills, and attitudes toward collaboration, resulting in the enhancement of clinical processes and patient care improvement as measured by adherence and satisfaction [6,7,8,9,10]. Interventions for promoting interprofessional collaboration (IPC) and team performance have increased exponentially such as, simulation training, interprofessional training, and the use of practical tools (clinical rounds, debriefing checklists, and technology) [11,12,13]. Studies have shown that implementing interprofessional collaboration interventions improved social functioning and limitations in physical activity [14] among older adults with cardiovascular diseases and improvement in pain control among older adults in palliative care [15].

Primary health care is an essential health service and is considered one of the cornerstones of universal health coverage [16], considering the role played by community health workers (CHWs) as community navigators, education providers, or as outreach agents [17]. Health care services in the Philippines are provided by a small number of health professionals (physicians, nurses, midwives, dentists, and sanitary inspectors) [18], and community health workers (CHWs) comprise the majority of each city’s human resource for health. Barangay health workers (community health workers) are employed in the local government units and are assigned to “barangays” (villages), the smallest administrative unit in the Philippines. Filipino CHWs are employed and trained by the city to assist in specific health programs such as health education, immunization, maternal and child health, and community profiling [19]. They are known to have the primary responsibility for channeling and implementing government programs, especially within the underserved populations [20]. Therefore, developing competencies among CHWs for collaboration is significant for enhancing their roles in interprofessional healthcare teams. 

Studies on interprofessional education (IPE) interventions have reported positive outcomes, including changes in learner perceptions/attitudes toward IPC and the value of collaboratively working with healthcare teams [16,17]. Though IPE and IPC improve cohesion and collaborative work within healthcare teams, the existence of soloing between different personnel, organizations, and care settings restricts formal IPC implementation [21]. The tension arising from a display of negative attitudes toward other professions contributes to poor communication among health care professionals and leads to dissatisfaction with teamwork [22]. IPC can be effective over a long period of time and as a result of practice-based training [23], which allows health professionals to cut old habits or stereotypes by acquiring new knowledge, skills, and attitudes [24]. Therefore, formal training of CHWs in IPC practice is needed for the promotion of interprofessional values and the improvement of knowledge regarding IPC. Considering the role of CHWs in planning and implementing health programs in the general population, it is important to educate and train them regarding IPC practice to prepare them to deliver care in collaboration with other health workers from diverse professional backgrounds. 

In light of this, and considering the lack of evidence on IPC initiatives in the Philippines, the objective of this study was to evaluate the efficacy of an in-service, short-term training program in improving the attitudes toward, and readiness and activities for collaboration among CHWs in a primary care setting in the Philippines.

## 2. Materials and Methods

### 2.1. Study Setting and Subjects

This study obtained data of community health workers through a longitudinal survey that was carried out as part of the training program conducted in the 50 barangays in urban cities in July 2019 in the Philippines. The cities included in the study were those that were engaged in health promotion activities and IPC practices, and offered various opportunities to CHWs for collaboration with the research team. The initial step was to obtain a mandate from the local government unit to conduct a pilot test of the in-service training program, followed by identifying the barangays that were providing services to at least 30 patients/residents who were 60 years or older on average, during a month. Barangays at each study site were then randomly assigned to either be an intervention or a control site. CHWs were recruited from these barangays to be a part of the training program. To be included, CHWs had to be directly involved in providing care to older adults aged 60 years or above, and to have been in their current occupation for more than one year at the time of the intervention. 

A total of 81 CHWs who met the inclusion criteria participated in the study. The sample size calculation for the quantitative aspect of this study was based on the expected difference between pre-test and post-test evaluation using the Readiness for Interprofessional Learning Scale (RIPLS) [25]. The dropout rate was 15%. CHWs belonging to the barangays that were randomly assigned as intervention sites participated in the intervention group (*n* = 42), and those from control study sites participated in the control group (*n* = 39). Those CHWs for whom either baseline or follow-up data were not available were excluded from the analysis.

### 2.2. Interprofessional Collaboration (IPC) Training Program

A three-day, competency-based, in-service interprofessional training program titled “Active learning to improve quality care: interprofessional collaboration (IPC) in gerontology services” was developed by a team of experts in public health, nursing, gerontology, primary healthcare, and IPC. 

The training program was composed of 10 modules focused on the theoretical perspectives of aging, health conditions, and care management in older age, comprehensive geriatric assessment, and IPC competencies integrated in the care of older adults. The objectives included gaining knowledge on healthy aging and age-associated conditions, performing a comprehensive geriatric assessment in collaboration with other health and social care workers, and defining individual roles and responsibilities as members of an interprofessional healthcare team. The program provided active learning opportunities and was composed of multiple teaching–learning strategies, including seminar-based discussions, role plays, video presentations, interprofessional group work, and reflection. 

The intervention consisted of three interactive learning days, eight hours a day, and with separate training programs organized per study site. Lessons-learned sessions were conducted at the end of each training day to build camaraderie and strengthen relationships between participants, along with a group discussion conducted at the end of the three-day training to identify changes needed in the workplace in anticipation of implementing IPC, the stakeholders/partners in IPC and gerontology care, and practical steps to enhance IPC with stakeholders.

### 2.3. Measurements

The outcomes of the study were the attitudes toward, and readiness and activities for collaboration among participants of the intervention and control groups prior to training (baseline) and six months after the training (follow-up). Independent variables include demographic and professional experience data such as age, years spent in current occupation, social support role, and study group (intervention or control). Other participant characteristics described are education, years from completion of formal education years of service in current practice and professional roles. The questionnaires used were the demographic information sheet, Attitudes Toward Health Care Teams Scale (ATHCTS) [26], Readiness on Interprofessional Learning Scale (RIPLS) [27], and Coordinated Activities Evaluation Scale (CAES) [28]. The reliability and validity of the ATHCTS, RIPLS, and CAES are reported elsewhere [26,27,28,29]. The questionnaires used were translated to the Filipino language to facilitate better understanding among participants.

Demographic Sheet. This was used to gather information regarding the independent variables in this study, such as age (20–39 years, 40–59, 60 or above), education (college undergraduate or college graduate), years passed since completion of formal education (1–5 years, 6–15, 16–25, 25 or more), and occupation (barangay health worker or barangay nutrition scholar). It also gathered information regarding their professional experience, such as years spent in the current occupation (1–5 years, 6–15, 16–25, 25 or more), years of service in the current workplace (1–5 years, 6–15, 16–25, 25 or more), and their roles and responsibilities (preventive, curative, home visit, social support, care coordination, or other). 

Attitudes Toward Health Care Teams Scale (ATHCTS). The ATHCTS consists of 21 items that are rated on a six-point Likert scale (1 = strongly disagree; 6 = strongly agree), and addresses patient outcomes as a result of team care, inefficiencies in teamwork, and equality among team members [26]. The minimum score is 6 and the maximum is 126. For high scores to reflect positive attitudes toward collaboration, coding was reversed for 9 negatively phrased items (items 2, 6, 9, 15, 16, 17, 19, 20, and 21). 

Readiness on Interprofessional Learning Scale (RIPLS). The RIPLS was used to evaluate participants’ readiness for and beliefs towards interprofessional learning. It consists of 19 items that are rated on a five-point Likert scale (1 = strongly disagree; 5 = strongly agree) [27], categorized under four domains that tackle aspects of teamwork and collaboration, professional identity, and roles and responsibilities [28]. The minimum score is 19 and the maximum is 95. To provide consistency and to ensure that high scores are interpreted as readiness or as positive attitudes for interprofessional learning, coding was reversed for 4 negatively worded items (items 10, 11, 12, and 18). 

Coordinated Activities Evaluation Scale (CAES). The CAES was used to evaluate activities for collaboration in the workplace. It consists of 15 items that are rated on a four-point Likert scale (0 = not at all; 3 = often) [29]. The minimum score is 0 and the maximum is 45, with a higher mean score indicating that activities for collaboration are present in the workplace.

### 2.4. Data Analysis

Descriptive analysis was used to analyze the demographic characteristics. Means and standard deviations were calculated for baseline and follow-up measurements of ATHCTS, RIPLS, and CAES. Additionally, mean differences were calculated to identify changes between baseline and follow-up among the two groups; a positive mean difference signified an increase in follow-up scores. A series of independent *t*-tests and one-way ANOVA were conducted to compare the mean scores of ATHCTS, RIPLS, and CAES according to participant characteristics and professional experience, and between intervention and control groups. Repeated measures analysis of variance was performed to compare the baseline and follow-up ATHCTS, RIPLS, and CAES mean scores of the control and intervention group. Partial eta squared ($\eta_{p}^{2}$) was used for effect size calculations in ANOVA, with all effects reported significant at *p* < 0.05. Multivariate linear regression analysis was also performed to identify associations between independent variables (age, education, years in current occupation, and social support role of CHWs) and key outcomes at baseline (mean differences of ATHCTS, RIPLS, and CAES). Statistical analyses were performed using SPSS Statistics for Windows, Version 25 (IBM Corp., Armonk, NY, USA). Statistical significance was set at *p* < 0.05.

### 2.5. Ethical Considerations

The study was approved by the World Health Organization Research Ethics Review Committee (ERC.003093), the Tokyo Medical and Dental University Ethics Review Board (M2017-232), and the Single Joint Research Ethics Board, Department of Health, Philippines (SJRED-2018-21). Participation in the study was voluntary and written informed consent was obtained from all participants before data collection.

## 3. Results

### 3.1. Participant Characteristics

A total of 81 CHWs responded to the ATHCTS, RIPLS, and CAES at baseline. Of the 81 CHWs, 42 belonged to the intervention group, while 39 were in the control group. Data for six participants were excluded (intervention, *n* = 5; control, *n* = 1), as there was no response during the follow-up survey. Thus, the final set of data used in the analysis included 75 CHWs (intervention, *n* = 37; control, *n* = 38), with a response rate of 92.6%. Table 1 presents participant characteristics disaggregated by group and the means and standard deviations of ATHCTS, RIPLS, and CAES scores at baseline according to participant characteristics. The majority of CHWs were 40 years or older (85.3%, Mean ± SD = 50.8 ± 9.6), and reported having spent 6 years or more in their current occupation (76.0%, 17.1 ± 10.3) and current workplace (66.7%, 12.9 ± 9.4). Social support (72.0%) and care coordination (73.3%) were the more commonly reported roles by the CHWs.

The overall mean scores of participants for ATHCTS, RIPLS, and CAES were 80.9 ± 6.3 (Mean ± SD), 79.5 ± 10.6, and 30.9 ± 7.5, respectively. CHWs who reported having spent more than 25 years in their current occupation showed a significantly higher ATHCTS score (Mean ± SD = 83.3 ± 7.8, *p* = 0.02) than those who had spent fewer years in their current occupation. As compared to those who were not engaged in social support roles, CHWs with social support roles had higher mean scores for ATHCTS, RIPLS, and CAES (81.8 ± 6.1; 79.8 ± 11.7; 31.2 ± 6.7), but only ATHCTS presented a significant difference (*p* = 0.02).

### 3.2. Comparison of ATHCTS, RIPLS, and CAES Scores within and between Groups

Table 2 shows the comparison of scores at baseline and follow-up, as well as the mean differences for both intervention and control groups. The intervention group presented a positive mean difference of ATHCTS (6.3 ± 8.3), while a negative mean difference was observed in the control group (−0.7 ± 8.2), significant at *p* < 0.01. Significant effects of ATHCTS were found for the intervention group ($\eta_{p}^{2}$ = 0.37, *p* < 0.001).

While a positive mean difference was observed for both groups on RIPLS score, the intervention group presented a higher mean difference, although no significant difference was observed (*p* = 0.48). In contrast to the positive mean differences on the ATHCTS and RIPLS in the intervention group, there was a negative mean difference observed in CAES scores in both the groups, indicating a decrease in mean scores during follow-up. A higher negative mean difference was also observed in the control group (−0.7 ± 7.7) as compared to the intervention group (−0.1 ± 8.1), at *p* = 0.67.

### 3.3. Association between Participant Characteristics and Survey Mean Differences

The multivariate linear regression analysis was performed to test whether participant characteristics predicted ATHCTS, RIPLS, and CAES scores difference at follow-up from baseline. The results are presented in Table 3. The intervention group presented an ATHCTS score increase at follow-up (Coefficient *β* = 6.17; 95% confidence interval (95% CI) = 0.82, 11.53; *p* = 0.03) as compared to the control group, after adjusting for age, years spent in current occupation, and social support roles. A positive association with the RIPLS mean difference (Coefficient *β* = 1.14; 95% CI = −6.52, 8.80; *p* = 0.77) and a negative association with CAES mean difference (Coefficient *β* = −1.56; 95% CI = −6.44, 3.31; *p* = 0.52) were observed but with no statistical significance.

## 4. Discussion

The present study evaluated the efficacy of an in-service short-term training program for impacting on the attitudes among CHWs in a primary care setting in the Philippines regarding caring for older adults, excluding the influence of age, years of current occupation of participants’, and their social support role in services. 

A comparison of mean differences between the two groups revealed that attitudes toward collaboration improved significantly in the intervention group as compared to the control group [26]. The difference in attitudes toward collaboration may have stemmed from training program experience and learning. Training is expected to improve health workers’ attitudes, knowledge, and skills about teams and team behavior [26] by utilizing multiple teaching–learning strategies [10,11,12,30]. This training program was implemented using role play, didactics, video presentations, and interprofessional group work activities, coupled with several opportunities for communication between health workers. The interprofessional group work activities were guided by a facilitator and allowed collaborative efforts on enumerating problems/concerns, diagnosis and treatment, community referral and rehabilitation procedures, and exchange of information and experiences [31]. As presented in the multivariate analysis, the intervention group showed significantly higher odds of achieving positive attitudes toward collaboration than those who did not participate in the training program, after adjusting for age, years spent in the current occupation, and the social support role played by them. Furthermore, participants in the intervention group reported that the training program provided knowledge about their roles and responsibilities, enabled them to become effective members of interprofessional teams, and provided an understanding of the goal of IPC, which is to achieve improved health outcomes.

Even though the ATHCTS mean score in the intervention group at baseline was significantly lower than that of the control group, the scores improved after the intervention. Previous IPE and IPC studies that utilized the ATHCTS to measure attitudes toward collaboration have presented mixed results. A longitudinal study that evaluated the effect of an IPE curriculum on students’ attitudes toward teamwork found no changes [32], whereas another study that examined the effects of the Geriatric Interdisciplinary Team Training Program reported significant improvements [33]; although both studies involved training programs that were implemented for a longer duration than the program implemented in this study. The ATHCTS had medium effect size [34] in improving attitudes toward collaboration, which indicates that the intervention improved attitudes toward collaboration among CHWs. Another study that evaluated the improvement in primary care professionals’ interprofessional attitudes after the implementation of an IPE program [35] reported an ATHCTS mean score of 69.8 ± 6.1 with item responses ranging from 0 (strongly disagree) to 5 (strongly agree). Since the present study used a rating scale from 1 to 5 to compare relative mean scores, each ATHCTS item of the present study was recoded to a zero base to create a total summed score ranging from 0 to 105. This, however, resulted in a mean score of 57.5, which is lower as compared to the score in the aforementioned study [35]. 

The total item mean score of RIPLS at baseline of the intervention and control groups was 4.19 (maximum score = 5), which is relatively higher than the total item mean score reported in a study conducted with health professional graduates in Germany, including nurses, therapists, and health care assistants [36]. The relatively higher RIPLS scores of Filipino CHWs in comparison to those of health professionals in another country setting, may indicate that the former had greater readiness for shared learning at baseline. Similarly, a relatively higher total mean score was evident in the CAES baseline scores within this study as compared to mean scores reported by a study conducted among Japanese community health professionals [29,37]. However, further analysis is needed to interpret the variation by population. 

In contrast to ATHCTS and RIPLS, the mean difference for the CAES showed a negative value, indicating that the follow-up scores were lower than the baseline scores. A systematic review of factors that enhance IPE program effectiveness revealed that capturing gradual change toward collaborative behavior requires programs of a longer duration coupled with periodical assessment [38]. In another study, the results of the content analysis revealed that collaboration was not evident among a number of primary care professionals after an IPE program, although other participants narrated otherwise [35]. Therefore, besides knowledge and skills, other factors likely influence collaborative behavior. In view of this, a time series evaluation might be more useful to objectively identify which IPC practices are affected by the training program. 

With respect to the number of years spent in their current occupation, CHWs who had been working for longer reported a relatively higher mean score in the ATHCTS than those who had worked for 5 years or less. This finding is supported by a previous study that examined how interprofessional experience affected graduate students’ attitudes toward interprofessional practice, and reported a positive association between years of interprofessional practice and attitudes toward teamwork after adjusting for demographic variables [38]. The results of the present study may be partly explained by the fact that older CHWs have more experience in interprofessional practice; therefore, they understand the significance of collaboration in achieving efficiency in work and better quality of patient care. 

Social support roles in this study include personal care, providing assistance in activity participation, transporting patients, and offering counselling and guidance on health behaviors. Those with social support roles had significantly higher positive attitudes toward collaboration at baseline than those who were not responsible for implementing social support interventions. However, multivariate statistical models presented a negative association between social support roles and positive attitudes toward collaboration after adjusting for age, group, and years in occupation. This can be explained by the fact that CHWs in social support roles may experience barriers in attending team meetings and, therefore, view collaboration as something that is time consuming, complicates their work, and hinders them from meeting the demands of their job [21,39].

The present study focuses on interprofessional collaborative practice, a concept that is still in its early conception in the Philippine health care system [18] and, at present, lacks documentation regarding existing practices and initiatives. Our study utilized standardized evaluation tools for measuring attitudes toward, and readiness and activities for collaboration in a healthcare setting. Despite the results indicating the efficacy of the training program, the study did have some limitations. First, changes in attitudes are self-reported. Second, the significant difference observed in baseline ATHCTS scores between the intervention and control groups could be attributed to the fact that the participants in the control group had more years of work experience in their current occupation. Third, there is limited generalizability of the results to CHWs from other geographical locations, considering variations in team practices from one city to another. Fourth, the questions on roles and responsibilities (social support and care coordination) were coded as either no or yes, which disables specific definitions of how these roles are being played out in the current occupation. 

The results of the study provide implications for educational and clinical practice. The efficacy of the training program implies the importance of planning for IPE with consideration to developing educators with an appropriate level of skills, knowledge, and clinical experience in geriatrics and institutional timetables to gather various health providers from different healthcare settings. IPE training for health providers must also integrate the learning context, relationship of the training content to prior learning, factors to improve teamwork and group balance, the teaching–learning strategies, and evaluation of outcomes. Educational practice must focus on preparing students for collaborative practice, formalizing several IPC practices such as team meetings where all health providers interact and learn. Recommendations include future studies to explore other predictor variables or contributors to positive attitudes toward collaboration such as beliefs, personalities, interprofessional practice experience, teaching–learning strategies for the delivery of IPC, and the association of collaboration competencies and improvement of health outcomes among older adults.

## 5. Conclusions

The results demonstrated the efficacy of a short-term, in-service training program on IPC in caring for older adults in improving attitudes of CHWs toward collaboration in healthcare teams. This training program, therefore, has the potential to be implemented in various health professions from different practice settings in countries with healthcare systems comparable to those in the Philippines. The interactive learning environment offered by the intervention coupled with multiple teaching–learning strategies and interprofessional group work possibly facilitated a significant increase in the ATHCTS mean score 6 months after the training, denoting positive attitudes toward collaboration. Furthermore, CHWs in the Philippines showed greater readiness for interprofessional learning and perceived the presence of collaborative activities in the workplace, which implies educational and clinical practice relevance of the training program in developing IPC competencies and, consequently, will prove helpful in achieving better health outcomes of older adults and communities.

## Figures and Tables

**Table 1:** ATHCTS, RIPLS, and CAES scores of community health workers at baseline (*N* = 75).

	All (*N* = 75)		ATHCTS (Range 21–126)	*p*	RIPLS (Range 19–95)	*p*	CAES (Range 0–45)	*p*
Characteristics	Intervention *n* (%)	Control *n* (%)	Mean ± SD		Mean ± SD		Mean ± SD	
All			80.9 ± 6.3		79.5 ± 10.6		30.9 ± 7.5	
Age				0.75		0.27		0.89
20–39	9 (24.3)	2 (5.3)	79.6 ± 9.0		83.2 ± 8.4		29.9 ± 6.7	
40–59	24 (64.9)	25 (65.8)	81.2 ± 6.0		79.7 ± 10.3		31.1 ± 8.4	
60 or above	4 (10.8)	11 (28.9)	80.9 ± 5.3		76.3 ± 12.8		31.0 ± 4.9	
Mean ± SD	48.7 ± 10.5	52.7 ± 8.4						
Education				0.15		0.50		0.06
College undergraduate	34 (91.9)	33 (86.8)	80.6 ± 6.2		79.2 ± 11.0		31.5 ± 7.7	
College graduate	3 (8.1)	5 (13.2)	84.0 ± 6.7		82.0 ± 7.1		26.3 ± 3.8	
Years passed since completion of formal education				0.35		0.52		0.22
1–5	0	1 (2.6)	90.0		73.0		26.0	
6–15	2 (5.4)	1 (2.6)	84.0		88.0 ± 8.7		39.7 ± 0.6	
16–25	5 (13.5)	6 (15.8)	80.1 ± 6.9		79.2 ± 16.8		33.1 ± 6.7	
>25	12 (32.4)	30 (78.9)	81.6 ± 5.7		80.0 ± 8.3		31.3 ± 7.8	
Mean ± SD	30.5 ± 9.6	32.5 ± 10.5						
Missing	18 (48.6)							
Years spent in the current occupation				0.02		0.96		0.40
1–5	10 (27.0)	1 (2.6)	75.6 ± 9.2		81.1 ± 6.4		28.6 ± 5.1	
6–15	13 (35.1)	8 (21.1)	81.8 ± 4.6		79.2 ± 12.9		32.1 ± 7.5	
16–25	8 (21.6)	13 (34.2)	81.6 ± 3.9		79.9 ± 9.0		30.1 ± 8.6	
>25	2 (5.4)	13 (34.2)	83.3 ± 7.8		78.9 ± 12.5		33.0 ± 6.8	
Mean ± SD	11.3 ± 8.4	22.6 ± 8.8						
Missing	4 (10.8)	3 (7.9)						
Years of service in current workplace				0.83		0.70		0.15
1–5	10 (27.0)	7 (18.4)	79.8 ± 9.9		77.9 ± 12.9		27.9 ± 4.7	
6–15	13 (35.1)	10 (26.3)	81.0 ± 4.8		80.5 ± 7.7		31.8 ± 9.0	
16–25	7 (18.9)	11 (28.9)	81.9 ± 4.6		80.6 ± 9.9		33.1 ± 6.4	
>25	2 (5.4)	7 (18.4)	80.7 ± 6.9		76.4 ± 15.5		32.4 ± 5.8	
Mean ± SD	11.0 ± 8.3	14.6 ± 10.1						
Missing	5 (13.5)	3 (7.9)						
Curative role				0.72		0.86		0.50
No	34 (91.9)	35 (92.1)	80.9 ± 6.4		79.5 ± 11.0		30.6 ± 7.5	
Yes	1 (2.7)	3 (7.9)	79.8 ± 5.1		78.5 ± 8.1		33.3 ± 5.9	
Missing	2 (5.4)							
Home visit role				0.34		0.06		0.94
No	9 (24.3)	8 (21.1)	79.7 ± 6.5		75.6 ± 13.8		31.1 ± 8.9	
Yes	26 (70.3)	30 (78.9)	81.3 ± 6.3		80.9 ± 9.1		30.9 ± 7.1	
Missing	2 (5.4)							
Social support role				0.02		0.65		0.40
No	16 (43.2)	3 (7.9)	77.9 ± 6.2		78.5 ± 7.6		29.5 ± 9.3	
Yes	19 (51.4)	35 (92.1)	81.8 ± 6.1		79.8 ± 11.7		31.2 ± 6.7	
Missing	2 (5.4)							
Care coordination role				0.22		0.33		0.83
No	11 (29.7)	7 (18.4)	79.3 ± 8.9		81.6 ± 7.0		30.4 ± 6.1	
Yes	24 (64.9)	31 (81.6)	81.4 ± 5.2		78.8 ± 11.7		30.9 ± 7.9	
Missing	2 (5.4)							
Other roles				0.41		0.67		0.43
No	32 (86.5)	31 (81.6)	80.6 ± 6.2		79.7 ± 10.7		30.5 ± 7.8	
Yes	3 (8.1)	7 (18.4)	82.4 ± 6.8		78.1 ± 11.8		32.5 ± 4.6	
Missing	2 (5.4)							
Group				<0.01		0.64		0.35
Intervention	37 (49.3)		78.4 ± 6.2		79.0 ± 10.2		30.2 ± 7.8	
Control	38 (50.7)		83.4 ± 5.5		80.1 ± 11.1		31.7 ± 7.3	

SD: Standard deviation; *p*: *p* value; ATHCTS: Attitudes Toward Health Care Teams Scale; RIPLS: Readiness on Interprofessional Learning Scale; CAES: Coordinated Activities Evaluation Scale.

**Table 2:** Mean difference of ATHCTS, RIPLS, and CAES scores between intervention and control groups.

	Intervention (*n* = 37)	Control (*n* = 38)	
	Mean ± SD	Mean ± SD	*p*
ATHCTS			
Baseline	78.4 ± 6.2	83.4 ± 5.5	<0.01
Follow-up	84.7 ± 5.7	82.3 ± 6.7	0.15
Difference	6.3 ± 8.3	−0.7 ± 8.2	<0.01
$\eta_{p}^{2}$	0.37	0.01	
* *p*	<0.001	0.58	
RIPLS			
Baseline	79.0 ± 10.1	80.2 ± 11.0	0.64
Follow-up	81.8 ± 6.9	80.8 ± 6.1	0.52
Difference	2.8 ± 11.0	0.8 ± 13.7	0.48
$\eta_{p}^{2}$	0.06	0.00	
* *p*	0.13	0.72	
CAES			
Baseline	30.2 ± 7.8	31.7 ± 7.3	0.40
Follow-up	30.2 ± 6.9	30.9 ± 7.8	0.69
Difference	−0.1 ± 8.1	−0.7 ± 7.7	0.67
$\eta_{p}^{2}$	0.00	0.01	
* *p*	0.97	0.56	

ATHCTS: Attitudes Toward Health Care Teams Scale; RIPLS: Readiness on Interprofessional Learning Scale; CAES: Coordinated Activities Evaluation Scale; $\eta_{p}^{2}$: partial ETA squared; * p: *p* value for repeated measures ANOVA.

**Table 3:** Multivariate associations between participant characteristics and mean differences on ATHCTS, RIPLS, and CAES.

	ATHCTS Mean Difference			RIPLS Mean Difference			CAES Mean Difference		
	Coefficient *β*	95% CI	*p*	Coefficient *β*	95% CI	*p*	Coefficient *β*	95% CI	*p*
Group									
Control	Ref			Ref			Ref		
Intervention	6.17	0.82, 11.53	0.03	1.14	−6.52, 8.80	0.77	−1.56	−6.44, 3.31	0.52
Age (in years)	−0.04	−0.30, 0.21	0.73	0.09	−0.27, 0.45	0.62	−0.09	−0.32, 0.14	0.45
Years spent in the current occupation	0.03	−0.24, 0.30	0.82	0.02	−0.37, 0.41	0.92	0.03	−0.21, 0.28	0.79
Social support role									
No	Ref			Ref			Ref		
Yes	−1.35	−6.68, 3.98	0.62	−0.23	−7.84, 7.39	0.95	−2.41	−7.26, 2.44	0.32

*β*: beta coefficient; ATHCTS: Attitudes Toward Health Care Teams Scale; RIPLS: Readiness on Interprofessional Learning Scale; CAES: Coordinated Activities Evaluation Scale.

## Data Availability

The data presented in this study are available on request from the corresponding author. The data are not publicly available due to participants’ confidentiality.
